# High Cell Density Fermentation of *Yarrowia lipolytica* on n-Hexadecane for the Valorization of Pyrolyzed Plastic Waste

**DOI:** 10.3390/ijms27021107

**Published:** 2026-01-22

**Authors:** Antonia Keil, Joost Woestenborghs, Oleksii Lyzak, Elodie Vlaeminck, Evelien Uitterhaegen, Karel De Winter, Kevin J. Verstrepen, Wim Soetaert

**Affiliations:** 1 Department of Biotechnology (BW25), Centre for Industrial Biotechnology and Biocatalysis (InBio.be), Faculty of Bioscience Engineering, Ghent University, Coupure Links 653, 9000 Ghent, Belgium; 2 Bio Base Europe Pilot Plant (BBEPP), Rodenhuizekaai 1, 9042 Ghent, Belgium; 3 Laboratory for Genetics and Genomics, Department of Microbial and Molecular Systems, Center of Microbial and Plant Genetics, KU Leuven, 3001 Leuven, Belgium; 4 VIB Laboratory for Systems Biology, VIB-KU Leuven Center for Microbiology, 3001 Leuven, Belgium

**Keywords:** *Yarrowia lipolytica*, high cell density, n-hexadecane, plastic pyrolysis, plastic recycling, triacylglycerols, citrate

## Abstract

The recycling of fossil-based plastic waste remains a key challenge in reducing environmental pollution and greenhouse gas emissions. An innovative approach is the biotechnological conversion of the n-alkane mixture obtained from thermal pyrolysis of plastic waste. This study focuses on the use of the oleaginous yeast *Yarrowia lipolytica* for the valorization of polyethylene (PE)-derived pyrolysis oil. From a screening of 50 *Y. lipolytica* strains, the most promising candidate was selected, and its single-cell phenotype was stabilized by *MHY1* deletion. In shake flask experiments, this strain grew similarly on 5–20 vol% of n-hexadecane, revealing no inhibitory effects. Subsequently, a high cell density fermentation was established in a 4 L bioreactor using a pulsed fed-batch approach, resulting in biomass concentrations of up to 145.6 g·L^−1^, which contained 22.0% triacylglycerols. In addition, cultivation at pH 2.5, compared to pH 4.0, reduced citrate formation from 95.6 to 0.8 g·L^−1^, while biomass and TAG titers remained similar. Overall, these results highlight the potential of integrating plastic waste-derived pyrolysis oil into future bioprocesses using *Y. lipolytica* as an effective platform for high cell density production.

## 1. Introduction

Today, fossil-based plastics are omnipresent in modern life and benefit from versatility, low-cost production, and widespread applicability. On the other hand, the environmental consequences of plastic waste accumulation have become apparent, which prompted intervention by the European Union through policies, like the European Green Deal. This led to an increase in overall recycling rates by more than 50% until 2020 compared to 2006 [1]. Despite this progress, the recycling rates of mixed plastic waste streams are 13 times lower than those of separated streams [1], and polyolefin wastes, such as polyethylene (PE), often undergo “downcycling” during mechanical recycling due to property loss [2]. An innovative and emerging chemical recycling method for such streams is pyrolysis, yielding a hydrocarbon mixture, comprising olefins and paraffins of various chain length [2,3]. A promising valorization strategy of such hydrocarbon mixtures is their utilization as fermentation feedstock, which was recently demonstrated at shake flask scale [4,5]. Indeed, the oleagineous yeast *Yarrowia lipolytica* shows the capacity to grow on paraffins and olefins from C_10_ to C_28_ [4,6,7] and even degrades complex hydrocarbon mixtures with potentially toxic impurities such as crude oil [8]. Furthermore, the availability of the *Y. lipolytica* genome, the established genetic toolbox [9], and its GRAS and QPS status [10] underscore its potential as a microbial cell factory for industrial fermentations targeting the valorisation of hydrocarbon mixtures derived from plastic waste.

Due to the immiscibility of hydrocarbons with water and the highly reduced state of the compounds, two main challenges for their effective use as a fermentation substrate arise: alkane availability and oxygen availability, respectively. To consume hydrocarbon substrates, *Y. lipolytica* naturally adapts by producing and excreting liposan as an emulsifier, in addition to producing hydrophobic protrusions on the cell surface [6,11]. Nonetheless, in the context of an industrial fermentation process where productivity and space-time yield are critical, overcoming limitations imposed by hydrocarbon substrate and oxygen availability in a bioreactor environment remains a key challenge. While the metabolic conversion of n-alkanes by *Y. lipolytica* has been investigated [6,11], the process performances achieved remain limited to around 25 g·L^−1^ cell dry weight (CDW) [12], and the key parameters governing the system at bioreactor scale have not been explored to date. Moreover, the use of increased concentrations of n-alkane substrates has not been considered due to suspected inhibitory effects on the used *Y. lipolytica* strain [12], whereas this could present a key strategy to achieve higher substrate availability.

Therefore, this study aims to achieve effective fermentation to high cell densities at the bioreactor level by overcoming the current barriers in substrate availability and conversion. We hypothesize that high cell densities can be reached on n-hexadecane in bioreactor fermentations through a *Y. lipolytica* screening selection process, yeast phenotype stabilization, and the application of a pulsed fed-batch method using high substrate concentrations. To identify a suitable strain, a broad panel of wild-type strains was first screened for growth on high concentrations of PE-derived pyrolysis oil as well as on pure hydrocarbons. The pyrolysis oil represents the target feedstock for future valorization, while the pure hydrocarbons are used as a mock stream to reduce feedstock complexity, allowing for better control of fermentation dynamics, especially at the bioreactor scale. For optimal performance in bioreactor fermentations, the most promising strain was further engineered by *MHY1* deletion, thereby preventing hyphae formation, which can hinder mixing and oxygen transfer [13]. Subsequently, this strain’s tolerance to elevated n-hexadecane concentrations was evaluated to define the operational limits for fed-batch cultivations. Finally, pulsed fed-batch fermentations were carried out in 4 L bioreactors, enabling close monitoring of key process parameters, aiming to achieve higher cell densities and to identify critical process challenges and key prospects for future developments.

## 2. Results and Discussion

### 2.1. Selection of Y. lipolytica Strain

**Figure 1:**
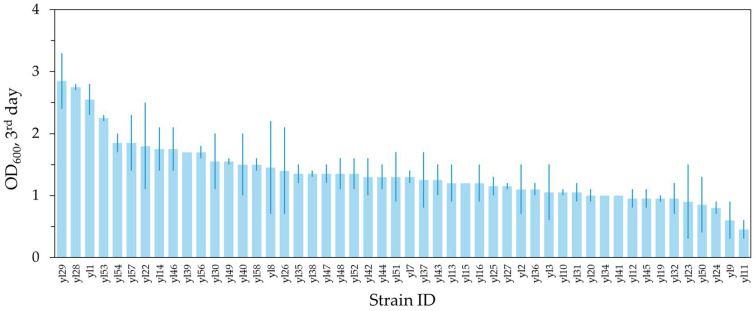
Final OD of strains from an in-house *Y. lipolytica* collection after growing for three days on SC + 20 vol% virgin PE pyrolis oil.

To find a suitable wild-type isolate for fermentation on pyrolysis oil, a screening of an in-house collection of 50 wild-type *Y. lipolytica* isolates was performed. To select isolates with optimal fitness, growth on and tolerance to pyrolysis oil for future broader applications, was assessed through Optical Density (OD) measurements. First, strains from the *Y. lipolytica* collection were tested for their ability to grow on pyrolysis oil obtained from the pyrolysis of virgin PE provided by the Sels group (CSCE, KU Leuven). Strains were grown in 24-well plates, 500 μL SC + 20 vol% pyrolysis oil for 3 days (starting OD = 1), after which the final OD was measured (Figure 1). The 22 fastest-growing strains, as well as the slowest strain as a negative control, were chosen for the next selection round.

**Figure 2:**
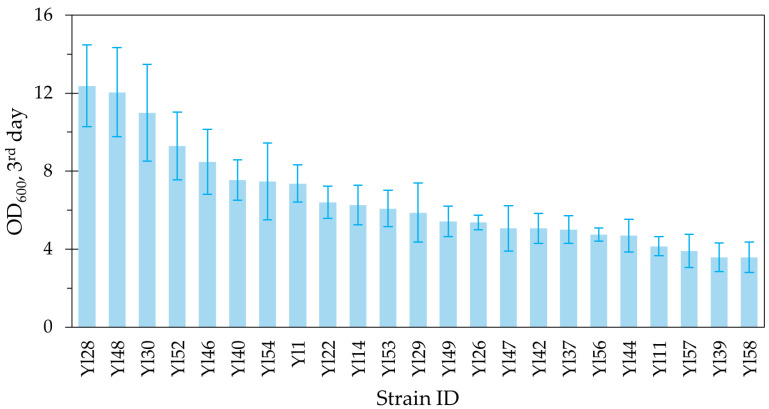
Average final OD of 23 strains after growing for three days on SC + 20 vol% hydrocarbon (n-octane, n-decane, n-dodecane, and n-hexadecane).

The next round of selection included testing the growth of 23 selected strains in 24-well plates, 500 μL SC + 20 vol% of four different pure hydrocarbon sources: n-octane, n-decane, n-dodecane, and n-hexadecane (starting OD = 1) (Figure 2). Using pure hydrocarbons reduces the complexity of the feedstock to better assess the ability of *Y. lipolytica* to grow on it as a carbon source. These four hydrocarbons are representative of the different lengths of linear hydrocarbons present in pyrolysis oil for future broader applications, whilst they are also safe to work with in lab conditions and readily available. The average OD after growing for three days on these four pure hydrocarbons was calculated as the next selection measure.

Finally, n-hexadecane was chosen as the main representative hydrocarbon length for pyrolysis oil because it is amongst the most prevalent carbon chain lengths in pyrolysis oil and is currently the most studied n-alkane feedstock for *Y. lipolytica*. Therefore, a final shake flask batch experiment was performed on the top eight performing strains in the previous screenings (Figure 2)—SC + 20 vol% n-hexadecane for up to 10 days (Table 1). The strain with the highest final OD was subsequently chosen for bioreactor-scale research.

Overall, strain Yl52 reached amongst the highest OD after three days on average on different pure hydrocarbons in 24-well-plates (Figure 2), and the highest final OD after 10 days on n-hexadecane in shake flasks (Table 1). One-way ANOVA with post hoc Tukey test confirms Yl52 performs significantly better than Yl30, Yl46, Yl48, and Yl54 but not significantly better than Yl1, Yl28, and Yl40. Therefore, Yl52 was deemed suitable for bioreactor-scale investigations of n-alkane-based fermentation processes.

### 2.2. Abolishing Filamentous Growth in Y. lipolytica Yl52 Through MHY1 Deletion

*Y. lipolytica* is a dimorphic organism. It can switch from single-cell to filamentous growth, forming hyphae [14]. The latter phenotype is considered undesirable in industrial applications as it hinders, amongst others, certain aspects of cultivation in bioreactors, such as mixing and oxygen transfer [13]. To abolish this, *MHY1* was deleted in strain Yl52. This gene encodes a transcription factor regulating the switch to filamentous growth. This deletion has been shown to completely abolish the transition to filamentous growth without hindering growth or stress tolerance [14].

The *MHY1* gene was deleted through homology-directed repair (HDR)-mediated genetic replacement of the *MHY1* coding sequence with hygromycin B resistance (*hphB*) cassette. After chemical transformation, colonies selected on Hygromycin B were checked through several colony PCRs for correct integration of the DNA through HDR into the locus of *MHY1*, thereby replacing the *MHY1* coding sequence. A subsequent chemical transformation of successful transformants introduced a replicative clonNAT plasmid with constitutive Cre recombinase expression to loop out the *hphB* resistance. Correct looping out of *hphB* was checked through both replica plating and colony PCR. Finally, after restreaking on regular Yeast Peptone Dextrose (YPD) medium, the replicative clonNAT plasmid was lost again, which was checked through replica plating. The final transformant was Sanger sequenced, showing a single loxP scar replacing the full coding sequence of *MHY1* as desired.

**Figure 3:**
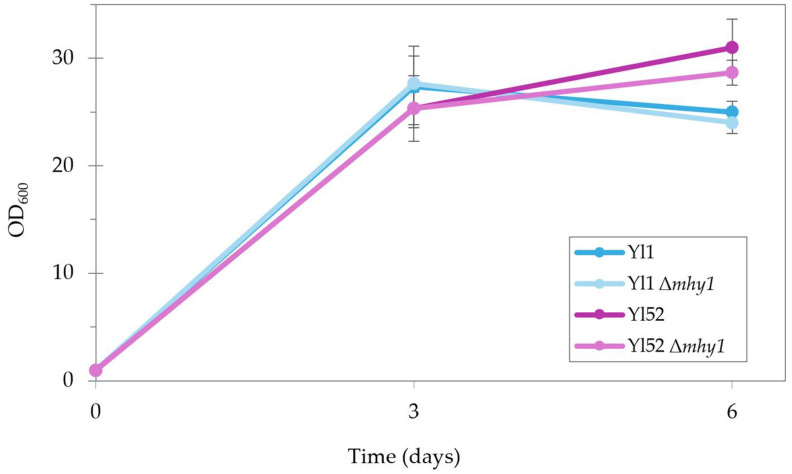
*Y. lipolytica* strains Yl1 (W29 *Y. lipolytica* reference lab strain) and Yl52 OD_600_ measurements over time of non-engineered vs. Δ*mhy1* on SC + 10 vol% n-hexadecane in shaking flasks.

To ensure that the deletion of *MHY1* has no effect on growth on medium with hydrocarbons as the carbon source, *Y. lipolytica* Yl52 and Yl1 (W29 *Y. lipolytica* reference lab strain) Δ*mhy1* transformants were grown in 20 mL in 100 mL flasks (starting OD = 1) in parallel with the non-engineered strain. OD measurements over time (Figure 3) showed no significant differences as expected, since previous studies confirmed the deletion of *MHY1* does not affect growth or stress tolerance in *Y. lipolytica* [14]. Thus, this confirms the *MHY1* deletion has no negative effect when *Y. lipolytica* is grown on n-hexadecane.

### 2.3. Robust Biomass Determination Method for Cultivation on Hydrocarbon Substrates

**Figure 4:**
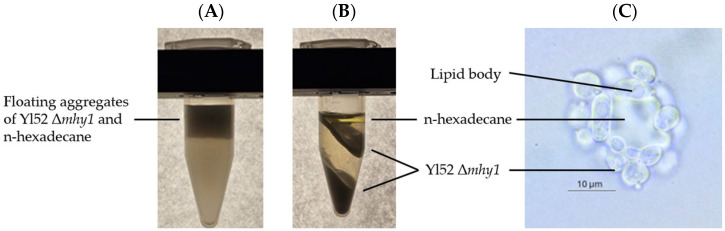
*Y. lipolytica* Yl52 Δ*mhy1* grown on n-hexadecane (**A**) settled for a few minutes, (**B**) centrifuged for 5 min at 14,200 rpm, and (**C**) microscopic image of n-hexadecane droplet surrounded by adsorbed cells.

The interaction of *Y. lipolytica* with the hydrophobic substrate during growth, i.e., by forming cell–substrate aggregates (Figure 4), imposes challenges on the gravimetric biomass quantification because the hydrocarbon substrate cannot be sufficiently separated from the aggregates (Figure 4), nor evaporated. For an in-depth study of the alkane fermentation process, beyond the preliminary, comparative screening of Section 2.1 and Section 2.2, a quantitative biomass determination method with improved robustness for hydrophobic substrates is required.

**Figure 5:**
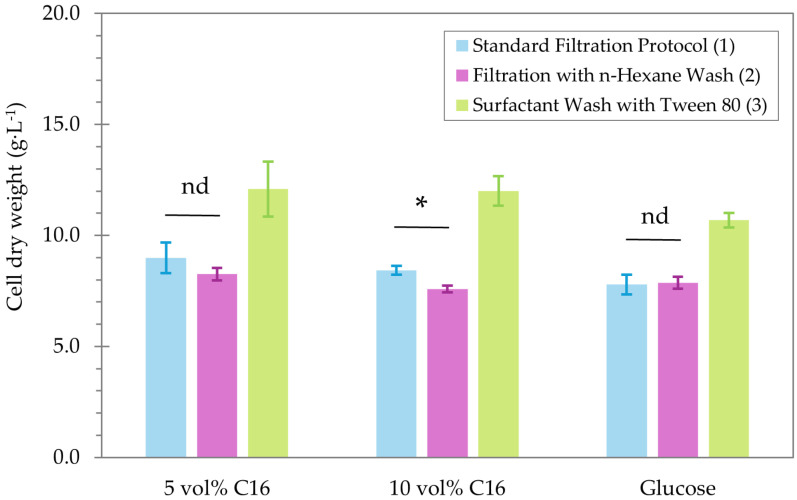
Cell dry weights (g·L^−1^) of *Y. lipolytica* Yl52 Δ*mhy1* obtained by Standard Filtration Protocol (1), Filtration with n-Hexane Wash (2), and Surfactant Wash with Tween 80 (3) grown on 5 and 10 vol% of hexadecane compared to glucose. Statistical differences were assessed with pairwise *t*-tests; the results are shown as not different (nd, *p* > 0.05) or significant (*, *p* < 0.05).

Three biomass determination methods were investigated, focusing on the separation of the cells from the hydrophobic substrate: (1) standard filtration protocol, (2) filtration with a short-chain alkane (i.e., n-hexane), and (3) a surfactant (i.e., Tween 80) wash (Table 2). The respective CDWs measured for *Y. lipolytica* Yl52 Δ*mhy1* grown for 24 h on 5 vol% and 10 vol% n-hexadecane were compared to CDWs measured for growth on glucose (i.e., without potential interactions of cells with the substrate) (Figure 5). The separation of biomass from the n-alkane substrate by means of a surfactant wash was effective; however, it resulted in an overestimation of the CDW as seen in the results for the glucose control. Likely, unbound Tween 80 remained with the biomass after washing and drying. The two filtration methods (1 and 2) resulted in the same CDW, confirming the cells are unaffected by the n-hexane wash (*p* = 0.891). On n-hexadecane, no significant difference was observed for the 5 vol% condition (*p* = 0.292), while a lower CDW was determined for the 10 vol% condition when the filter was washed with n-hexane (*p* = 0.016). This demonstrates that the standard filtration protocol is insufficient to remove the remaining n-hexadecane, which is still attached to the cells, and that an additional wash with a volatile hexadecane-solubilizing agent is required. Hence, the CDW filtration method, including the wash (2), was used for subsequent experiments.

### 2.4. Tolerance to High Concentrations of n-Hexadecane

**Figure 6:**
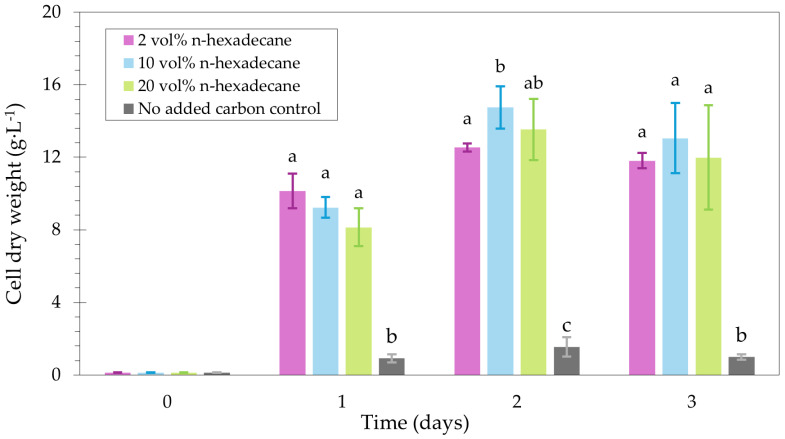
Cell dry weight (g·L^−1^) of *Y. lipolytica* Yl52 Δ*mhy1* grown on 2 vol%, 10 vol%, 20 vol% of n-hexadecane, and without a carbon source. Groups that share a letter are not significantly different; groups with different letters differ significantly (*p* < 0.05, Bonferroni-corrected α = 0.0167), assessed with pairwise *t*-tests for each day.

To investigate the tolerance of the selected *Y. lipolytica* strain to elevated n-hexadecane concentrations, the growth on various batch concentrations (2 vol%, 10 vol%, and 20 vol%) was investigated. For each condition, the CDW was determined daily for three days. The same biomass concentrations were reached regardless of the n-hexadecane start concentration on day one (Figure 6), which indicates a tolerance to up to 20 vol% of n-hexadecane. In the literature, batch concentrations beyond 5 g·L^−1^ (0.65 vol%) n-hexadecane have not been considered in bioreactor cultivations [15], potentially due to an earlier report observing no growth on 10 g·L^−1^ (1.29 vol%) n-hexadecane [16]. In contrast, the current study demonstrates the absence of growth inhibition at higher n-hexadecane concentrations for *Y. lipolytica* Yl52 Δ*mhy1*. The latter may have been enabled by the following approach. Indeed, through the *Y. lipolytica* broad strain screening and selection at elevated concentrations of PE-derived pyrolysis oil and hydrocarbon mixtures, a tolerant strain could be identified and used as a high-potential starting point for further research. As a result, the use of elevated n-alkane substrate concentrations is enabled, and restrictions of previous approaches related to limited n-alkane substrate supply can be overcome, allowing the investigation of intensified biomass production from n-hexadecane at bioreactor scale.

### 2.5. Overcoming Substrate Availability Limitations at Bioreactor Scale

**Figure 7:**
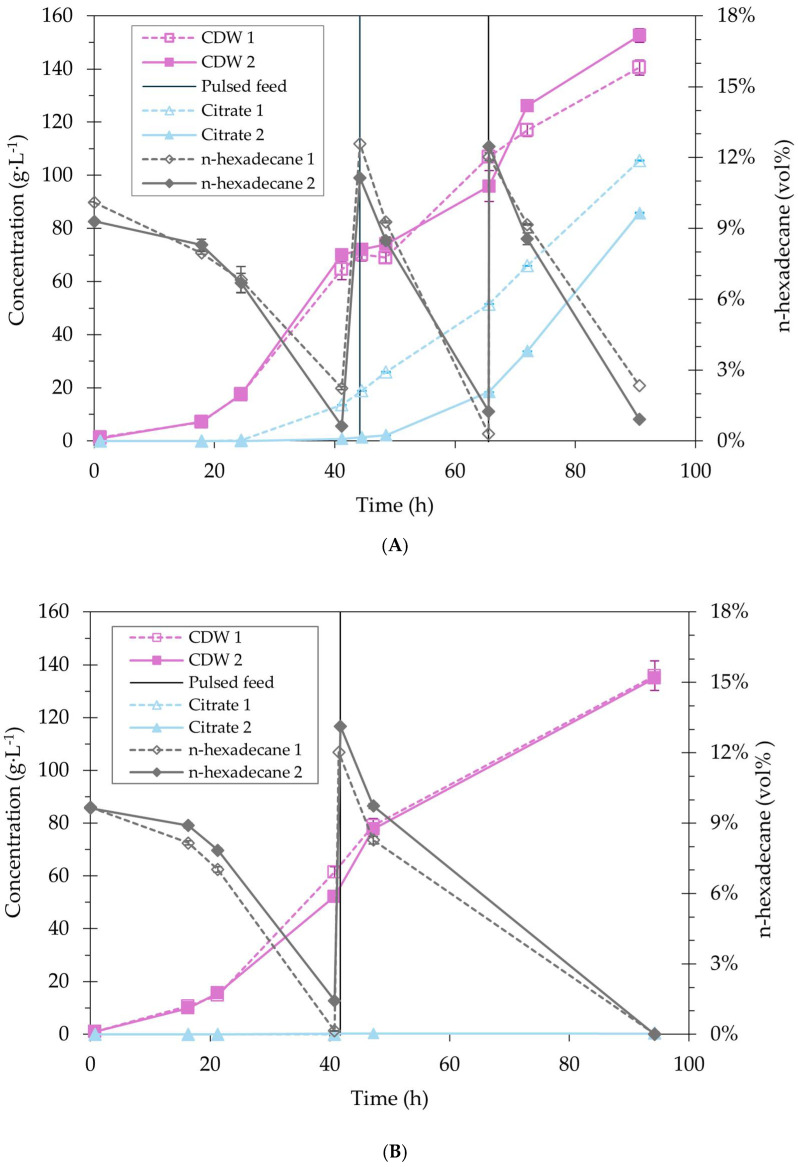
Cultivation of *Y. lipolytica* Yl52 Δ*mhy1* at pH 4.0 (**A**) and pH 2.5 (**B**) in 4 L bioreactors: biomass concentration g·L^−1^ (CDW), n-hexadecane (vol%), citrate concentration g·L^−1^. Vertical lines indicate pulsed feedings of 150 mL of n-hexadecane.

To study the potential of n-hexadecane fermentations with *Y. lipolytica* Yl52 Δ*mhy1* towards high cell density, the process was transferred to a bench-scale bioreactor with continuous monitoring of process parameters. The cultivation was performed using fed-batch pulsed feedings, with a total addition of 450 mL of n-hexadecane over 90.7 h, resulting in a final biomass concentration of 145.6 ± 8.5 g·L^−1^ (Figure 7A). This is the first report achieving high cell densities with *Y. lipolytica* on an n-alkane substrate, representing a more than five times higher biomass concentration achieved in the same time [12]. The overall consumption rate of 3.37 ± 0.01 g·L^−1^·h^−1^ n-hexadecane represents a more than 15-fold improvement over previously reported values [15]. Notably, the increased consumption rate persists after biomass normalization, indicating two underlying causes or a combination of them: firstly, an improvement of n-hexadecane availability and secondly, an increased assimilation efficiency of the used strain. The former is linked to the higher n-hexadecane volumetric ratio, resulting in an increased interfacial area for cell attachment, and an increased probability of cell-droplet encounters [17]. Adverse effects of using higher volumetric ratios, i.e., coalescence effects, can be avoided at 10 vol% [17].

**Figure A1:**
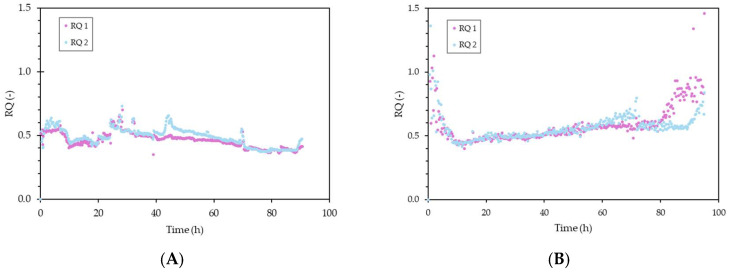
Respiratory quotient (RQ) (-) of the cultivations of *Yarrowia lipolytica* Yl52 Δ*mhy1* at pH 4.0 (**A**) and pH 2.5 (**B**) in 4 L bioreactors.

To evaluate the oxygen availability during the fermentation, the dissolved oxygen (DO) and off-gas composition were monitored. The DO dropped to 0% after around 24 h in all fermentations, marking the onset of oxygen limitation. This high O_2_ demand was further reflected in the respiratory quotient (RQ), defined as the ratio of CO_2_ emitted to O_2_ consumed, which dropped to below 0.50 (Figure A1 in Appendix A). Notably, based on stoichiometry, assuming complete conversion of n-hexadecane into biomass, an RQ of 0.51 would be expected. The lower values in the later stages of the fermentation indicate that a portion of the carbon is diverted to overflow metabolites, primarily citrate, rather than biomass. Compared with glucose, the conversion of n-hexadecane to biomass requires 53.1% more oxygen and emits 34.7% less CO_2._ Although the high oxygen requirements pose challenges for n-alkane-based processes, recent studies showed that n-hexadecane acts as an oxygen vector with the potential to increase the oxygen transfer rates by 90% in shake flasks [18,19]. Hence, future work focusing on more detailed characterization of oxygen supply requirements under varying mixed n-alkane concentrations could further elucidate their impact on oxygen transfer, substrate consumption, and potential economic implications.

Next to biomass, 95.7 ± 13.9 g·L^−1^ of citrate were accumulated, which is associated with overflow metabolism during the stationary growth phase [20], likely related to the onset of oxygen limitation in this study. A nutrient limitation, particularly nitrogen limitation, was not observed in this study, which is known to trigger citrate production [20,21,22]. A known strategy to prevent this is the selection of a pH set-point below the lower pK_a_ of citrate of 3.13 [23,24], which reduces the transport across the cell membrane.

To investigate the potential of cultivating *Y. lipolytica* Yl52 Δ*mhy1* on n-hexadecane at a highly acidic pH set-point of 2.5, a second fermentation was carried out. Indeed, citrate accumulation substantially reduced to 0.83 ± 0.63 g·L^−1^ (Figure 7B). However, due to acidic stress, growth and n-hexadecane consumption rates were reduced (Table 3). Despite this, high cell densities of 135.4 ± 0.7 g·L^−1^ were reached in 94.3 h, highlighting the benefit of adding high volumetric ratios of n-hexadecane for increased substrate availability. However, excessive foaming and gas hold-up impeded reliable sampling and mixing after 50 h until n-hexadecane was fully consumed. Therefore, the affected samples were removed from Figure 7B, and no second pulsed feeding was carried out. At pH 4, such effects were not observed, leading to the conclusion that a pH of 2.5 results in intensified emulsion stability. This is potentially related to the characteristics of the produced emulsifier, which would require further investigation.

The final TAG content of the cells was determined for the fermentations to assess the final biomass composition. Similar TAG titers of 32.3 ± 2.2 g·L^−1^ (22.0 ± 0.2 wt%) and 36.7 ± 0.46 g·L^−1^ (27.1 ± 0.5 wt%) were reached in the fermentations at pH 4.0 and pH 2.5, respectively (Table 4). During growth on n-hexadecane, the production of TAGs occurs ex novo; thus, not requiring a nutrient limitation in contrast to de novo production [25,26]. TAG accumulation by *Y. lipolytica* of up to 50% CDW has been demonstrated [6]. Through further process development, e.g., steering nutrient supply [27] to enhance the TAG content of the cells, cost competitiveness could be improved.

## 3. Materials and Methods

**Figure 8:**
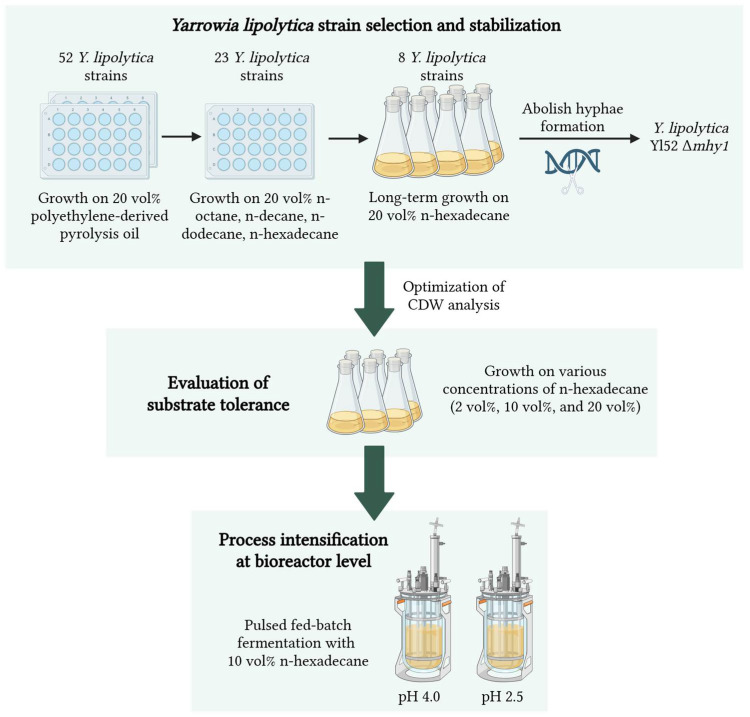
Methodological approach (Created in BioRender. Keil, A. (2026) https://app.biorender.com/illustrations/695cf0b62ce106c5603fc29d?slideId=6fecd32e-9cb4-4b61-a818-a954e188ba0e (accessed on 12 January 2026)).

An overview of the followed methodological approach is depicted in Figure 8.

### 3.1. Screening

In-house collection of *Yarrowia lipolytica* was streaked from −80 °C on YPD agar (20 g·L^−1^ bactopeptone, 10 g·L^−1^ yeast extract, 20 g·L^−1^ D-glucose, 20 g·L^−1^ agar). Cells of *Y. lipolytica* from an agar plate were inoculated in 24-well plates in 500 µL YPD medium (20 g·L^−1^ bactopeptone, 10 g·L^−1^ yeast extract, 20 g·L^−1^ D-glucose), and grown overnight at 30 °C at 900 rpm. The next morning, the cells were washed with growth test medium (1.9 g·L^−1^ yeast nitrogen base, 12.5 g·L^−1^ (NH_4_)_2_SO_4_, and 0.79 g·L^−1^ complete supplement mixture) and inoculated in 24-well plates in 500 µL growth test medium containing 20 vol% selected hydrocarbon feedstock, with an initial OD_600_ of 1. The cells were incubated at 30 °C and 900 rpm.

Screening in 250 mL non-baffled shake flasks was performed in 20 mL of the same medium as before. Cells were grown overnight at 30 °C in 5 mL YPD medium in 15 mL falcon tubes in a rotating wheel. Shake flasks were incubated at 30 °C and shaken at 200 rpm.

### 3.2. Yeast Chemical Transformation

The *MHY1* gene was deleted using an adapted chemical transformation protocol from Abdel-Mawgood & Stephanopoulos (2020) [28]. Donor DNA used in this transformation was an *Escherichia coli* plasmid containing the donor template to replace the *MHY1* coding sequence. This template consisted of 1.5 kb homology regions upstream and downstream of the *MHY1* coding sequence, with the *hphB* resistance gene inserted between two loxP sites instead of the *MHY1* coding sequence. Colony PCR checks were performed using SapphireAmp fast PCR and checked on 1% agarose gel electrophoresis. Selection media used are YPD agar + 150 µg·mL^−1^ Hygromycin B, or +250 µg·mL^−1^ nourseothricin (ClonNAT). Successful final mutants were grown overnight in YPD and frozen in 25% glycerol stocks at −80 °C. Final Sanger sequencing of Δ*mhy1*::*loxP* was performed on GXL PCR amplicon (size verified by 1% agarose gel electrophoresis) by Eurofins Genomics EU (Ebersberg, Germany).

### 3.3. Cultivation Conditions

Cryovials of *Yarrowia lipolytica* Yl52 Δ*mhy1* were prepared by mixing 0.75 mL of culture grown overnight on YPD (10 g·L^−1^ yeast extract (YE), 20 g·L^−1^ peptone, and 20 g·L^−1^ glucose (VWR, Radnor, PA, USA)) with 0.75 mL of 40 *w*/*v*% glycerol in reverse osmosis (RO) water. The cryovial was used to inoculate 250 mL baffled shake flasks with 50 mL YPD medium and incubated at 28 °C and 175 rpm. After 18–20 h, 6 mL were transferred to 141 mL YNB medium (4.2 g·L^−1^ yeast nitrogen base without amino acids and without ammonium sulfate, 5 g·L^−1^ (NH_4_)_2_SO_4_, and 3 g·L^−1^ Hy-Yest 412 yeast extract (YE) (Tralee, Ireland)) set to a pH of 5.5, and 3 mL hexadecane (>95% purity, Thermo Fisher, Waltham, MA, USA). The cells were incubated at 28 °C and 160 rpm, and 75 mL of the culture was used to inoculate one bioreactor. For the experiment in Section 2.4, the cells grown on YPD were washed twice with YNB medium without a carbon source to remove leftover glucose from the previous seed step. The 500 mL baffled shake flasks were filled with 58.5 mL of YNB, including 0, 2, 10, or 20 vol% of n-hexadecane, and inoculated with 1.5 mL of this solution to reach a start OD_600_ of 0.3. 10 mL of the sample was taken per day.

### 3.4. Bioreactor Cultivation

The cultivations were carried out in 4 L Eppendorf DASGIP^®^ glass reactors (Hamburg, Germany) at 1.5 L filling volume. The medium for the bioreactor contained 4.2 g·L^−1^ YNB without amino acids and without (NH_4_)_2_SO_4_, 5 g·L^−1^ (NH_4_)_2_SO_4_, 3 g·L^−1^ YE, and 5 g·L^−1^ KH_2_PO_4_. All compounds, except for the yeast extract, were dissolved in RO water by adjusting the pH to 2.5–3.0 and filter sterilized. The YE was autoclaved in the reactor for 90 min at 121 °C and 1 atm. The n-hexadecane was also filter sterilized. Concentrations were calculated based on the total volume, including the start concentration of 10 vol% n-hexadecane. The pH during the fermentation was maintained at pH 4.0 or pH 2.5 with 24.5% NH_4_OH and 2 M H_3_PO_4_. The DO was maintained at 30% air saturation by cascade control of the stirring speed (400–1400 rpm) and manual increases in the air flow (28 L·h^−1^–180 L·h^−1^). The temperature was maintained at 28 °C. The off gas composition was measured using GC-MS. Fermentations were carried out as independent biological duplicates.

### 3.5. Sampling and Analytical Methods

The samples of the bioreactor fermentations were stirred for the distribution into different vessels for alkane quantification due to immediate unmixing without stirring. A total of 1 mL of broth was frozen per sample.

### 3.6. Biomass Methods

The OD was measured at 600 nm wavelength using the Cell density meter Ultrospec10 (Amersham Biosciences, Little Chalfont, UK).

For the filtrations with (2) and without (1) short-chain alkane wash, a Glass Vacuum Filtration Device, Diameter ø 47 mm/50 mm (Sartorius, Göttingen, Germany) with cellulose acetate filter papers 410 of VWR^®^ was used. Filtered sample volumes were between 1.5 and 3.5 mL, depending on the cell density. The filters were washed with 5 mL RO water, and in case of (2), washed with n-hexane or n-heptane with >95% purity afterwards. To dry, filters were left overnight in the fume hood, to dry and the weight was determined with an analytical balance.

For the surfactant wash (3), 50 µL of polysorbate 80 was added to 3.5 mL of broth and vortexed for 2 min. After centrifugation for 5 min at 14,200 rpm, the supernatant was discarded. The cell pellet was resuspended in 5 mL of distilled water once and then suspended in 5 mL of distilled water. The washed broth was poured onto a glass fiber filter and dried at 105 °C in the moisture analyzer MA37 (Sartorius, Göttingen, Germany).

CDW was measured as a technical triplicate for the method development (Section 2.3) and the fermentations (Section 2.5) and as a technical duplicate for the shake flask test (Section 2.4).

### 3.7. Alkane Extraction and Quantification

n-Hexadecane was extracted with n-heptane (>99.5% purity, 1:2 *v*/*v* n-heptane/broth) containing 75 mg·L^−1^ deuterated n-hexadecane (98% D) as the internal standard. GC analysis was carried out on a Trace DSQ from Thermo Fisher equipped with a TRACE DSQ MS Detector 70 L·s^−1^. The samples (1 µL) were injected with a Thermo PAL GC-xt (PAL, Zwingen, Switzerland) autosampler in split mode with a split ratio of 50:1 at an inlet temperature of 230 °C. The used column is a Rxi-5Sil MS GC Capillary Column (30 m, 0.25 mm ID, 0.25 µm). The oven temperature program was as follows: 5 min at 35 °C, heat up to 230 °C at 10 °C·min^−1^, and followed by a 4 min hold time, and a heat-up to 300 °C at 10 °C·min^−1^ followed by a 1 min hold time. Helium was used as a carrier gas. The used software is Xcalibur (version 3.0). The volume of hexadecane was calculated from the measured concentration with the following equation:

$$V_{C16}=\frac{c_{C16}\times {\;V}_{C7}}{\rho_{C16}-c_{C16}}$$

With $V_{C16}$: volume of n-hexadecane (L), $c_{C16}$: concentration of n-hexadecane in n-heptane (g·L^−1^), $V_{C7}$: volume of n-heptane (0.5 × 10^−3^ L), $\rho_{C16}$: density of n-hexadecane (773 g·L^−1^) at 25 °C.

The calibration curve was in the range from 25 mg·L^−1^ up to 250 mg·L^−1^ with an R^2^ of at least 0.995. The limit of detection (LOD) is 0.03 mg·L^−1^ and the limit of quantification (LOQ) is 0.11 mg·L^−1^. Measurements below the LOQ were indicated, and samples above the highest concentration were diluted to fall within the calibration curve.

The results of GC-MS quantification were combined with the weight-based determination of fed n-hexadecane. When n-hexadecane had been fully consumed prior to sampling, the exact time-point of consumption was determined with the aid of the off-gas analysis for the calculation of productivities or consumption rates.

### 3.8. Citrate Quantification

The samples for HPLC were filtered through a 0.2 µm PES filter. The quantification of citrate was performed using an Agilent 1260 Infinity High-performance Liquid Chromatography (HPLC) (Santa Clara, CA, USA) equipped with a Metacarb 67H column (300 × 6.5 mm, connected to a varia 5244GC precolumn) with a refractive index detector (RID). 0.8 mL min^−1^ of 2.5 mM H_2_SO_4_ was flown through the column, which was kept at 40 °C. The results were processed with Agilent OpenLab CDS (version 3.6). Samples were measured in duplicate. A calibration curve in the range of 0.2 to 25.0 g·L^−1^ was established with an R^2^ of above 0.999. The LOD is 0.054 g·L^−1^, and the LOQ is 0.162 g·L^−1^. Measurements below the LOQ were indicated, and samples above the highest concentration were diluted to fall within the calibration curve.

### 3.9. TAG Derivatization and Quantification

A total of 200 µL of MilliQ water and 300 µL of 12 M HCl were added to 100 µL of broth and mixed thoroughly. The vials were incubated for 1 h at 80 °C and 1500 rpm. When the vials had cooled down to room temperature, 300 µL of methanol and 300 µL of butyl acetate, including 524 mg·L^−1^ methyl-undecanoate as the internal standard, were added. After thorough mixing, the samples were centrifuged at 10,000 rpm for 15 min. 50 µL of the upper layer was transferred to a new vial and mixed with 350 µL of 2.5% sulfuric acid in methanol (*v*/*v*). After another heating cycle at 80 °C and 1500 rpm for 1 h, the samples were left to cool down to room temperature before adding 500 µL 1 M NaCl and 250 µL butyl acetate with internal standard. After vortexing and settling, the upper layer was transferred to a new vial and analyzed in the GC-MS. The instrument, as described in “alkane extraction and quantification” was used. Here, a split of 100:1 was used for the injection. The oven temperature program was as follows: 5 min at 40 °C, heat up to 230 °C at 10 °C/min, followed by a 4 min hold time, and a heat-up to 300 °C at 10 °C/min, followed by a 1 min hold time. The protocol and analysis were carried out in triplicate for each sample. The calibration curve range, R^2^ values, LOQs, and LODs (calculated from signal-to-noise ratios of 3 for LOD and 10 for LOQ) for each fatty acid methyl ester are reported in Table 5. The sum of all fatty acid methyl esters is reported as the total TAG concentration. The samples were diluted 18 times during the derivatization protocol and further diluted as needed when measurements fell outside the calibration curve.

## 4. Conclusions

This study presents the first high cell density fermentation of *Y. lipolytica* on n-alkane feedstocks. This was achieved by selecting a strain that grows effectively on crude PE-derived pyrolysis oil and the selected model n-alkane: n-hexadecane, even at elevated concentrations of 20 vol%. This allowed the usage of high volumetric ratios of n-hexadecane at bioreactor scale to overcome previous limitations of substrate availability. Overall, the results highlight the substantial potential of using n-hexadecane-containing feedstocks, such as PE-derived pyrolysis oil, in bioprocesses with *Y. lipolytica*, as evidenced by the achieved high cell densities. In the follow-up research, the effect of the pyrolysis oil composition resulting from the presence of dyes, stabilizers, or additives in the plastic waste should be addressed. In addition, TAGs emerge as a promising target product, which can be used for the production of biodiesel and oleochemicals. Future bioprocess optimization, potentially combined with targeted strain engineering strategies, could further steer product profiles to improve yields of specific compounds and eventually increase process efficiency towards economic feasibility.

## Figures and Tables

**Table 1:** Final mean OD ± standard deviation of the top eight selected strains growing on SC + 20 vol% n-hexadecane.

Strain	OD After 10 Days
Yl1	30.33 ± 1.70
Yl28	27.00 ± 2.94
Yl30	17.33 ± 2.05
Yl40	30.00 ± 2.16
Yl46	20.67 ± 4.50
Yl48	20.67 ± 1.25
Yl52	33.67 ± 1.25
Yl54	20.67 ± 5.25

**Table 2:** Overview of tested CDW determination methods.

	Standard Filtration Protocol (1)	Filtration with n-Hexane (2)	Surfactant Wash (Tween 80) (3)
0.	-	-	Addition of 50 µL of Tween 80 to 3.5 mL of broth and vortexed for 2 min
1.	Filtration of 3.5 mL broth	Filtration of 3.5 mL broth	Centrifugation and decantation
2.	Wash with 5 mL RO water	Wash with 5 mL RO water	Wash with 5 mL RO water
3.	-	Wash with 5 mL n-hexane	Wash with 5 mL RO water
4.	Dried	Dried	Dried

**Table 3:** Overall residual biomass yield ($Y_{X/S}$), maximum growth rate ($\mu_{max}$), overall residual biomass productivity ($Q_{X,\;overall}$), overall n-hexadecane consumption rate ($r_{S,overall}$), peak n-hexadecane consumption rate ($r_{S,peak}$), and n-hexadecane consumption rate normalized to the average biomass concentration ($q_{S,avg}$) of the two pulsed fed-batch cultivations at pH 4.0 and 2.5 with *Y. lipolytica* Yl52 Δ*mhy1*.

pH	$Y_{X/S}$	$\mu_{max}$	$Q_{X,overall}$	$r_{S,overall}$	$r_{S,peak}$	$q_{S,avg}$
	$g\cdot g^{-1}$	$h^{-1}$	$g\cdot L^{-1}\cdot h^{-1}$	$g\cdot L^{-1}\cdot h^{-1}$	$g\cdot L^{-1}\cdot h^{-1}$	$g\cdot g^{-1}\cdot h^{-1}$
4.0	0.37 ± 0.02	0.14 ± 0.00	1.26 ± 0.07	3.37 ± 0.01	5.57 ± 0.04	0.18 ± 0.05
2.5	0.45 ± 0.00	0.08 ± 0.01	1.04 ± 0.01	2.50 ± 0.15	5.07 ± 0.11	0.11 ± 0.00

Data is the average ± the standard deviation of two biological replicates.

**Table 4:** Final concentrations of TAGs ($c_{TAGs}$) and of citrate ($c_{Cit})$, overall productivities of TAGs ($Q_{TAGs}$) and citrate ($Q_{Cit}$), and overall yields of TAGs ($Y_{TAGs/S})$ and citrate ($Y_{Cit/S}$) of the two pulsed fed-batch cultivations at pH 4.0 and 2.5 with Y. lipolytica Yl52 Δmhy1.

pH	$c_{TAGs}$	$c_{Cit}$	$Q_{TAGs}$	$Q_{Cit}$	$Y_{TAGs/S}$	$Y_{Cit/S}$
	$g\cdot L^{-1}$	$g\cdot L^{-1}$	$g\cdot L^{-1}\cdot h^{-1}$	$g\cdot L^{-1}\cdot h^{-1}$	$g\cdot g^{-1}$	$g\cdot g^{-1}$
4.0	32.3 ± 2.2	95.7 ± 13.9	0.36 ± 0.02	1.07 ± 0.16	0.11 ± 0.01	0.31 ± 0.04
2.5	36.7 ± 0.5	0.82 ± 0.63	0.39 ± 0.00	0.01 ± 0.01	0.17 ± 0.00	0.00 ± 0.00

Data is the average ± the standard deviation of two biological replicates.

**Table 5:** Lowest and highest concentration of the calibration curve, R^2^, LOQ, and LOD for each fatty acid methyl ester.

Fatty Acid Methyl Ester	Lowest Calibration Concentration	Highest Calibration Concentration	R^2^	LOQ	LOD
	mg·L^−1^	mg·L^−1^	-	mg·L^−1^	mg·L^−1^
C15:0	1.89	27.77	0.994	0.09	0.03
C16:1 (cis)	5.37	307.78	0.999	1.53	0.46
C16:0	10.86	204.59	0.998	0.25	0.07
C18:1 (cis)	23.62	367.41	0.994	0.28	0.09
C18:2 (cis)	16.61	376.99	0.999	0.53	0.16
C18:3 (alfa)	9.42	103.78	0.994	0.12	0.03
C18:0	5.73	255.31	0.999	0.19	0.06
C24:0	4.80	24.00	0.999	0.60	0.18

## Data Availability

The raw data supporting the conclusions of this article will be made available by the authors on request.

## Abbreviations

The following abbreviations are used in this manuscript:

CDW	Cell dry weight
DO	Dissolved oxygen
GRAS	Generally Recognized As Safe
HDR	Homology-Directed Repair
HPLC	High-performance Liquid Chromatography
LOD	Limit of detection
LOQ	Limit of quantification
OD	Optical density
PCR	Polymerase Chain Reaction
PE	Polyethylene
QPS	Qualified Presumption of Safety
TAG	Triacylglycerol
YE	Yeast extract
YNB	Yeast nitrogen base
YPD	Yeast peptone dextrose

## Appendix A

Stoichiometric equations on n-hexadecane and glucose towards biomass as the sole product.

$$0.11\;C_{16}H_{34}+1.53\;O_{2}\to 1\;{CH}_{1.8}O_{0.5}+1\;H_{2}O+0.78\;CO_{2}$$

$$0.37\;C_{6}H_{12}O_{6}+1.00\;O_{2}\to 1\;{CH}_{1.8}O_{0.5}+1.30\;H_{2}O+1.20\;CO_{2}$$
